# Review: Local Tumor Necrosis Factor-α Inhibition in Inflammatory Bowel Disease

**DOI:** 10.3390/pharmaceutics12060539

**Published:** 2020-06-11

**Authors:** Bahez Gareb, Antonius T. Otten, Henderik W. Frijlink, Gerard Dijkstra, Jos G. W. Kosterink

**Affiliations:** 1 Department of Clinical Pharmacy and Pharmacology, University Medical Center Groningen, University of Groningen, Hanzeplein 1, 9713 GZ Groningen, The Netherlands; j.g.w.kosterink@umcg.nl; 2 Department of Pharmaceutical Technology and Biopharmacy, Groningen Research Institute of Pharmacy, University of Groningen, Antonius Deusinglaan 1, 9713 AV Groningen, The Netherlands; h.w.frijlink@rug.nl; 3 Martini Hospital Groningen, Department of Clinical Pharmacy and Toxicology, Van Swietenplein 1, 9728 NT Groningen, The Netherlands; 4 Department of Gastroenterology and Hepatology, University Medical Center Groningen, University of Groningen, Hanzeplein 1, 9713 GZ Groningen, The Netherlands; a.t.otten@umcg.nl (A.T.O.); gerard.dijkstra@umcg.nl (G.D.); 5 Department of PharmacoTherapy, -Epidemiology and -Economics, Groningen Research Institute of Pharmacy, University of Groningen, Antonius Deusinglaan 1, 9713 AV Groningen, The Netherlands

**Keywords:** inflammatory bowel disease, tumor necrosis factor-α, local, topical, site-specific, drug targeting, antibody, antisense, miRNA, prokaryote, eukaryote

## Abstract

Crohn’s disease (CD) and ulcerative colitis (UC) are inflammatory bowel diseases (IBD) characterized by intestinal inflammation. Increased intestinal levels of the proinflammatory cytokine tumor necrosis factor-α (TNF-α) are associated with disease activity and severity. Anti-TNF-α therapy is administered systemically and efficacious in the treatment of IBD. However, systemic exposure is associated with adverse events that may impede therapeutic treatment. Clinical studies show that the efficacy correlates with immunological effects localized in the gastrointestinal tract (GIT) as opposed to systemic effects. These data suggest that site-specific TNF-α inhibition in IBD may be efficacious with fewer expected side effects related to systemic exposure. We therefore reviewed the available literature that investigated the efficacy or feasibility of local TNF-α inhibition in IBD. A literature search was performed on PubMed with given search terms and strategy. Of 8739 hits, 48 citations were included in this review. These studies ranged from animal studies to randomized placebo-controlled clinical trials. In these studies, local anti-TNF-α therapy was achieved with antibodies, antisense oligonucleotides (ASO), small interfering RNA (siRNA), microRNA (miRNA) and genetically modified organisms. This narrative review summarizes and discusses these approaches in view of the clinical relevance of local TNF-α inhibition in IBD.

## 1. Introduction

Ulcerative colitis (UC) and Crohn’s disease (CD) are immune-mediated types of inflammatory bowel diseases (IBD) affecting the gastrointestinal tract (GIT). IBD is a chronic disease with a course characterized by remission and relapse. Disease symptoms include chronic diarrhea, abdominal pain, weight loss and bloody stools. The severity combined with the chronic nature of the disease results in a decreased health-related quality of life, disability and frequent hospitalizations. Whereas the continuous and diffuse inflammation of the mucosa in UC is typically limited to the rectum and may extent proximally, the granulomatous transmural inflammation in CD affect most commonly the ileo-colonic region [1,2,3].

**Figure 1:**
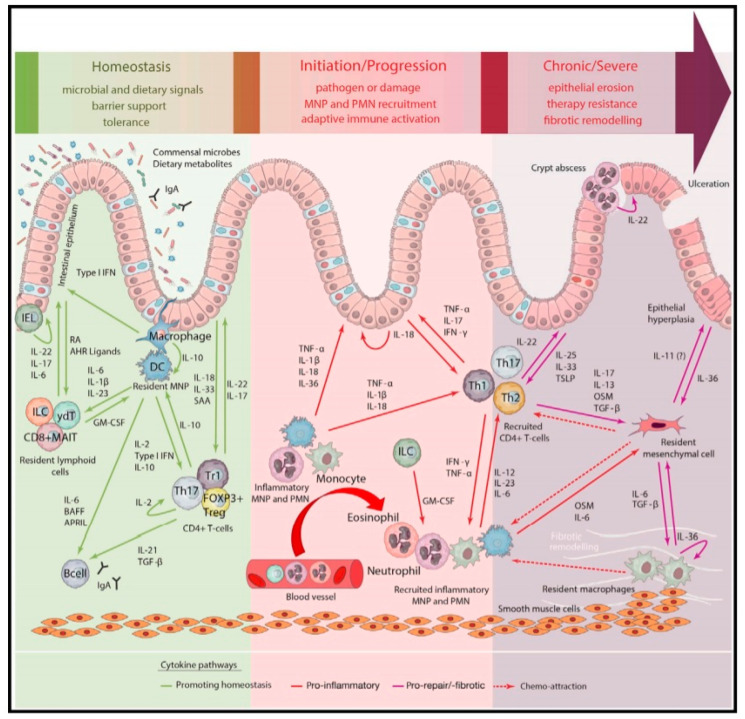
Mucosal immunology of the gastrointestinal tract (GIT) under homeostasis, acute inflammation and chronic inflammation in inflammatory bowel diseases (IBD). The aberrant immunological response of the innate immune system induces an acute inflammatory state that may progress to chronic inflammation with a prominent role of the adaptive immune system. The involved cytokine network is complex and shows a major role of the proinflammatory cytokine tumor necrosis factor-α (TNF-α). For an explanation and overview of all the abbreviations, the reader is referred to the original work of this Figure [4]. Reprinted from Friedrich et al. [4] with permission from Elsevier.

Although the exact pathogenesis of IBD is unclear, research shows that a combination of genetics, environmental factors and the microbiome play a prominent role in the onset of gut epithelial dysfunction. Consequently, increased exposure of the gut wall to luminal antigens trigger an aberrant acute inflammatory response driven by the innate immune system. Secretion of proinflammatory cytokines such as interleukin (IL)-1β, IL-6 and tumor necrosis factor-α (TNF-α) results not only in tissue damage, but the activation of the adaptive immune system as well. Tissue damage in turn may result in an increased exposure of the gut wall to luminal antigens inducing a stronger activation of both the innate and adaptive immune system, which perpetuates the inflammatory state resulting in chronic inflammation (Figure 1) [2,3,4,5,6,7].

TNF-α is a pleiotropic proinflammatory cytokine implicated in a wide range of cellular processes including cell proliferation, survival and death. In addition, TNF-α signaling is associated with the regulation of several inflammatory pathways including the cyclooxygenase-2 (COX-2) and inducible nitric oxide synthase (iNOS) pathways [8,9,10,11]. Hence, TNF-α is a key mediator in the inflammatory response. TNF-α is predominantly secreted by monocytes, macrophages and natural killer cells [12,13,14,15,16]. TNF-α is first synthesized as a transmembrane protein (tmTNF-α) and can induce immunological responses in effector cells, but also transduce reverse signaling by contact-dependent cell interactions. In addition, tmTNF-α can be enzymatically cleaved by TNF-α-converting enzyme (TACE) resulting in soluble TNF-α (sTNF-α). Upon distribution in the extracellular space or systemic circulation, sTNF-α may exert immunological effects at distant sites. Therefore, both forms are active cytokines that share similar as well as distinctive immunological effects. TNF-α activation of effector cells under physiological conditions generally leads to a proinflammatory response or apoptosis and aids in the defense against infections and localized tissue injury [12,13,14,15,16]. However, the elevated TNF-α tissue levels in the mucosa and lamina propria of IBD patients result in an aberrant proinflammatory response that is associated with the dysregulation of mucosal immune cells and tissue damage [4,7].

**Figure 2:**
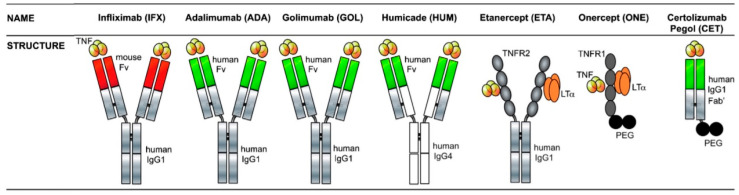
Overview of anti-TNF-α biologicals relevant for IBD. Humicade is CDP571. Abbreviations: Fab’—antigen-binding fragment; Fv—variable fragment; Ig—immunoglobulin; LTα—lymphotoxin-alpha; PEG—polyethylene glycol; TNF—tumor necrosis factor-α; TNFR—TNF receptor. Reprinted from Sedger et al. [16] with permission from Elsevier.

Anti-TNF-α therapy aims to antagonize the effects of TNF-α. Examples of anti-TNF-α therapies which are or have been used in the clinical setting of IBD are infliximab (IFX), adalimumab, golimumab, certolizumab, etanercept, onercept and CDP571 (Figure 2). These biologicals are antibodies or soluble TNF-α receptors (sTNFR) that neutralize TNF-α. Although the main mechanism of action is TNF-α antagonism, these drugs have distinctive pharmacodynamic profiles that are specific for the individual compound partly due to the variations in the molecular structure. Hence, the observed efficacy of the different anti-TNF-α therapies in IBD vary and are not equivalent (reviewed in: [13,17,18,19,20,21,22]). The desired therapeutic effects include a sustained anti-inflammatory response, mucosal healing and restoration of the gut epithelial barrier function [23,24]. However, anti-TNF-α therapy is associated with adverse events related to systemic exposure. These adverse events include infusion reactions [25,26], psoriasis or psoriasiform lesions [27], osteonecrosis of the jaw [28,29], the development of antinuclear antibodies (ANA) [30,31,32,33] and an increased risk of opportunistic infections [34,35,36] and developing lymphoma [37]. Additionally, infusion reactions are associated with therapy discontinuation [38]. Systemic administration may induce anti-drug antibodies (ADA), which in turn is associated with infusion reactions as well as loss of efficacy [39,40,41].

Research shows that the local immunological environment in the GIT correlates with IBD disease activity [42,43,44,45], type [46,47,48] and relapse [49,50]. Furthermore, studies investigating the local immunological environment of the GIT before and after anti-TNF-α therapy show that the therapy reduces histological and endoscopical disease activity [51,52,53], inhibits activation of immune cells [54,55,56], downregulates the expression of cell adhesion molecules and proinflammatory cytokines [53,57,58,59,60,61,62], modulates apoptosis of monocytes as well as enterocytes [63], restores gut barrier function [64,65] and levels of antimicrobial peptides [66] and has a favorable effect on the gut microbiome [67,68,69]. Importantly, it was shown that anti-TNF-α therapy induces a potent local, but not a systemic effect [70] and that gut tissue concentrations may correlate better with a clinical and sustained response compared to serum levels alone [71,72]. This may partly explain anti-TNF-α therapy failure despite therapeutic drug concentrations. Collectively these observations suggest that local as opposed to systemic TNF-α inhibition may be an efficacious treatment option for IBD which may have fewer adverse events related to systemic exposure. However, major challenges in accomplishing site-specific TNF-α inhibition with macromolecules such as proteins are drug targeting and the subsequent stability of the drug in the GIT. More important, drug penetration into the targeted inflamed sites is a prerequisite for drug efficacy, but may arguably impose the biggest challenge since the absorption mechanisms and kinetics of macromolecules differ substantially from smaller chemical entities [73,74,75,76,77,78].

The objective of this narrative review was to evaluate the available literature on PubMed with regards to local TNF-α inhibition in IBD. First, animal studies investigating the efficacy or feasibility of local TNF-α inhibition in IBD are discussed. These studies investigated formulations containing antibodies, antisense oligonucleotides (ASO), small interfering RNA (siRNA), microRNA (miRNA) and genetically modified organisms. Subsequently, clinical studies ranging from case reports to randomized placebo-controlled clinical trials that investigated the efficacy or feasibility of local TNF-α inhibition in IBD are discussed. This review aims to summarize the available literature on local TNF-α inhibition with macromolecules intended for the treatment of IBD.

## 2. Methods

This is a narrative review. However, in view of finding relevant citations in the medical literature, the following search strategy was used on PubMed. The search term for citations with regards to local biologic therapy was ”(tumor necrosis factor OR TNF OR tumor necrosis factor inhibitor OR TNF inhibitor OR anti-tumor necrosis factor OR anti TNF OR infliximab OR adalimumab OR certolizumab pegol OR golimumab OR etanercept OR onercept OR humicade OR CDP571) AND (local OR locally OR tissue OR intralesional OR intralesionally OR site specific OR direct OR directly OR topical OR topically OR targeted OR target OR targeting OR rectal OR rectally OR enema OR suppository OR oral OR orally OR colonic OR colon OR ileum OR ileo-colonic OR mucosa OR mucosal) AND (Crohn’s disease OR inflammatory bowel disease OR ulcerative colitis OR proctitis OR pancolitis OR colitis OR IBD)”. This yielded 8339 hits, published from June 1985–May 2020. The citations published up to 1 May 2020 were included in this review.

The search term for citations with regards to gene-silencing therapy was “(gene silencing OR antisense OR RNA silencing OR small interfering RNA OR siRNA OR oligonucleotide OR antisense oligonucleotide) AND (tumor necrosis factor OR tumor necrosis factor-alpha OR TNF) AND (Crohn’s disease OR inflammatory bowel disease OR ulcerative colitis OR proctitis OR pancolitis OR colitis OR IBD)”. This yielded 400 hits, published between July 1993–May 2020. The citations published up to 1 May 2020 were included in this review.

All the titles and abstracts of the citations were read. The reference sections of the included citations were read for additional relevant citations that could be included in this review. Citations that investigated local TNF-α inhibition in human or animal studies were included. Studies that investigated systemically administered anti-TNF-α therapy with experiments aimed to elucidate the local effects in gastrointestinal (GI) regions were included as well. Citation that conducted in vitro studies without any in vivo investigations were excluded.

Out of a total of 8739 citations, 31 animal studies are summarized in Table 1 and discussed in Section 3. Preclinical studies on local TNF-α inhibition and 17 clinical studies are summarized in Table 2 and discussed in Section 4. Clinical studies on local TNF-α inhibition. Section 3. predominantly reviews experimental therapy and formulation strategies that aim to target the localized inflammation sites in the GIT in IBD animal models. Section 4. reviews the available clinical studies that investigated the efficacy or feasibility of local TNF-α inhibition in IBD. In view of readability we used the word ‘significant’ to depict a ‘statistically significant effect’ whereas ‘significant’ was not used to discuss experiments that showed a remarkable effect without statistical significance or on which no statistics were performed at all by the authors of the original citations.

## 3. Preclinical Studies on Local TNF-α Inhibition

### 3.1. Considerations

Anti-TNF-α formulations have been investigated in animal models (Table 1). These animal models were predominantly chemically induced colitis models in mice though some studies investigated the formulations in genetically induced colitis models or healthy animals. The most commonly used colitis models for IBD research were dextran sulfate sodium (DSS)- and 2,4,6-trinitrobenzenesulfonic acid (TNBS)-induced chemical colitis models, which share resembles with UC and CD, respectively [79,80].

Several antibodies were investigated in IBD animal models in the context of local TNF-α inhibition. These antibodies were or were not produced by a host carrier. For instance, prokaryotic or eukaryotic carriers of a vector that produce anti-TNF-α antibodies may secrete the antibody in the GIT of the host in view of local TNF-α inhibition. Alternatively, the carrier may be used to deliver a vector to gut epithelial cells that express the protein after genetic transformation. These complex processes impose great challenges in order to achieve reproducible and therapeutic local TNF-α inhibition since drug levels are dependent on many factors that are variable such as the host microbiome, carrier growth rate, transformation efficiency, drug expression rate by the carrier or transformed host cells and drug stability in the GIT. These factors may be subjected to inter- and intraindividual fluctuations as a result of the dynamic GI environment and in turn correlate with fluctuations in efficacy.

Nucleotide formulations have been investigated as well. The investigated formulations were ASO, siRNA, miRNA or chemical modifications thereof to increase the stability and/or efficacy. ASO are single-stranded nucleotides that are typically 10–50 nucleotides long whereas siRNA are typically 15–25 nucleotides long. Both can modulate gene expression by a variety of mechanisms which are out of the scope of this review. Simplified and generally speaking, ASO can bind to complementary pre-mRNA or mRNA and alter splicing or induce degradation by endogenous RNase H, respectively, whereas siRNA binds to endogenous RNA-induced silencing complex and thereby induces mRNA degradation. Both approaches aim to silence target genes (reviewed in: [74,81,82,83,84,85]). However, miRNA are endogenously produced small, non-coding RNA strands of typically 20–25 nucleotides long that are implied in several cellular and gene regulation processes (reviewed in: [86,87]).

Targeted cytoplasmic nucleotide delivery is a prerequisite for gene silencing. To deliver nucleotides to targeted cells, the formulation must protect the nucleotides from environmental degradation, aid in targeted cellular uptake by endocytosis, and must facilitate endosomal escape of the nucleotides into the cytoplasm [73,82,83]. These processes can be influenced by different approaches and formulation strategies of which several are discussed in this review. However, besides targeting the drug to the site of inflammation, these processes add jet another major challenge for drug efficacy due to the complexity of these mechanisms. Furthermore, the released drug concentration at the site of inflammation may not always correlate with intracellular drug concentrations. The complexity of targeted ASO is depicted by mongersen, an orally administered ASO against Smad7 aimed to restore transforming growth factor-beta (TGF-β) signaling. The phase II clinical trial results [88] were encouraging whereas the phase III clinical trial showed no significant efficacy [89]. The investigators stated that no mucosal drug concentrations were measured during the phase III trial, which may have partly explained the observed ineffectiveness. Therefore, strategies to evaluate the effective delivered dose in animal as well as clinical studies are of great value for oligonucleotide therapy.

### 3.2. Antibodies

The efficacy of rectally administered IFX (IFX-enema) compared to IV administration was investigated in a mouse model of acute DSS colitis [90]. As expected, IV IFX (5 mg/kg) showed a significant effect in reducing loss of bodyweight, loss of colon length and disease activity index (DAI). These effects were similar for 300 µg rectally administered IFX. Furthermore, histopathologic analysis showed a marked decrease in inflammation of both treatment groups compared to control. Interestingly, analysis of IFX in serum, colonic mucosa, and stools showed that the levels in serum and colon were significantly lower in colitic mice compared to healthy mice in the IV treatment group. However, IFX levels in stool were remarkably higher in colitic mice compared to healthy mice. An explanation for this may be the loss of IFX via ulcerated epithelial surfaces in stools, resulting in the low in vivo concentrations. This phenomenon has been reported in UC patients [91]. These results show that the efficacy of rectally administered IFX is comparable to IV IFX in a mouse model of acute DSS colitis.

V565 is a 115 amino acid 12.6 kDa single domain antibody [92]. In vitro results showed that V565 neutralized sTNF-α and tmTNF-α with a comparable efficacy as adalimumab. In GI simulation studies, V565 stayed active with no remarkable loss of activity after 2 h, 2 h and 16 h incubation in mouse small intestine supernatants, human ileal fluids and human fecal extracts, respectively. Moreover, no substantial loss of activity was observed after 6 h incubation with the digestive enzymes trypsin, chymotrypsin and pancreatin. However, all activity was lost after 2 h incubation with pepsin. In vivo results in healthy mice confirmed these observations since active V565 could be measured in the stomach, small intestine, caecum and colon during GI transit. In a DSS colitis model, V565 could penetrate the colonic mucosa and submucosa whereas no noticeable penetration in healthy colons was observed, which indicates that orally administered antibodies are able to penetrate the inflamed regions in vivo. Interestingly, serum levels of V565 could be detected in colitic mice, but not in healthy mice. In addition, colon concentrations correlated with serum concentration in colitis mice. These results indicate that V565 could be absorbed during colitis, presumable by the enhanced permeability and retention effect of the inflamed colon [93]. Though no in vivo effectiveness study was performed, ex vivo experiments with human IBD tissue showed that V565 could inhibit the phosphorylation of several signaling proteins implied in the proinflammatory response.

The same research group [94] investigated V565 formulated in a tablet coated with the pH-sensitive polymer Eudragit L100 (pH-threshold ≥6) [95]. This coating was applied for the time-dependent drug release in the ileum, cecum, colon and rectum. Dissolution experiments showed that V565 was released in a sustained manner after 2 h at pH ≥ 6. In vivo experiments in cynomolgus monkeys showed that the formulation disintegrated in the small intestine and reached parts of the lower colon. However, GI transit time and pH values of these monkeys [96,97] may not be representative of those seen in humans [98,99,100,101,102,103]. Measured fecal concentrations indicated that V565 transited through the GIT after the coating disintegrated. Serum concentrations of V565 were also observed in this study, showing that V565 is partly absorbed after oral administration. V565 has been investigated in a clinical study [104] and is discussed in Section 4.

AVX-470 is an orally administered antibody against TNF-α derived from the colostrum of cows that have been immunized with TNF-α [105]. The in vitro potency of AVX-470 is comparable to IFX. In a prophylactic acute DSS and TNBS colitis model, mice were given AVX-470 in doses of 1–10 mg/day before the induction of colitis. Endoscopy scores in both colitis models showed significant improvement with a trend towards a dose-dependent relationship. Furthermore, the efficacy was comparable with prednisolone or etanercept [106] in a chronic colitis model. In this model, TNF-α and proinflammatory cytokines mRNA were significantly reduced (~50%). These findings were mirrored by histopathologic experiments, which showed that AX-470 penetrated predominantly in the lamina propria, mucosa and muscularis mucosa region of inflamed, but not healthy colon of mice. As with V565, this observation show that orally administered antibodies penetrate the inflamed sites of the colon in vivo [94]. However, systemic exposure after oral treatment was low to non-existing, demonstrating the site-specific effect of this formulation. AVX-470 has been investigated in a clinical study [107,108] and is discussed in Section 4.

Avian-anti-TNF-α is an oral formulation containing polyclonal anti-TNF-α antibodies derived from the yolks of immunized hens [109]. An in vitro experiment confirmed the TNF-α neutralizing potency of the antibody. Moreover, significant effects on colon weight, myeloperoxidase (MPO) activity, histopathology scores and colon morphology scores were seen in an acute TNBS colitis model in rats after oral treatment with 600 mg/kg/day. In this same colitis model, Avian-anti-TNF-α was compared with oral sulfasalazine (200 and 1000 mg/kg/day) or dexamethasone (2 mg/kg/day). The efficacy of Avian-anti-TNF-α was comparable with these treatment groups. The efficacy was further investigated in a chronic colitis model and these results also showed significant effects on colon weight, histopathology scores and colon morphology scores, though no comparison with other drugs was investigated in the chronic colitis model. Histological analysis showed that Avian-anti-TNF-α could be detected in the lamina propria, muscularis mucosa and submucosa of ulcerated sites of the colon, further emphasizing that orally administered antibodies are able to penetrate the inflamed colonic regions in vivo [94,105].

### 3.3. Antisense Oligonucleotides

ISIS 25302 is an ASO targeting murine TNF-α. In the first animal study investigating the efficacy of SC doses ranging from 0.25–12.5 mg/kg, a dose-dependent decrease in disease severity and colonic TNF-α mRNA expression was reported [110]. Another animal study in *db/db* mice, known for the expression of TNF-α in their adipose tissue [111], showed a significant reduction of TNF-α mRNA (64%) expression after IPadministration [112]. Furthermore, in an acute DSS colitis model significant effects on colon length and DAI was observed after multiple IV dose of 1 mg/kg. These effects were comparable with mice treated with 25 µg anti-TNF-α antibody. A trend towards a linear dose–effect correlation was observed in a chronic colitis model with doses ranging from SC 0.25–12.5 mg/kg, showing significant effects on DAI, histopathology scores and colonic TNF-α mRNA levels. These results were comparable with 15 µg anti-TNF-α antibody. In another chronic colitis model of IL-10^−/−^ mice investigating prophylactic as well as therapeutic treatment regimens with SC doses ranging from 0.01–10 mg/kg, reductions in histopathology scores and TNF-α as well as interferon-gamma (IFN-γ) levels measured in colonic organ cultures were seen. Since ISIS 25302 was administered systemically in these studies, off-target, systemic anti-inflammatory effects contributing to the favorable response cannot be ruled out [110,112].

In a follow up study, ISIS 25302 was associated with galactosylated low molecular weight chitosan (Gal–LMWC–ASO) to form a nano-complex [113]. The galactose residues of Gal-LMWC have high affinity for macrophage galactose-type lectin (MGL), which is expressed on macrophages. MGL expression is enhanced during immune activation and facilitates receptor-mediated endocytosis [114]. In vitro results indeed showed a substantial increase in macrophagic transfection of Gal–LMWC–ASO compared with naked ISIS 25302. Furthermore, intracolonic administration of 5 mg ASO/kg showed that Gal–LMWC–ASO accumulated in the inflamed colon of mice with no remarkable accumulation in other organs. Interestingly, Gal–LMWC–ASO did not accumulate in healthy colon of mice, indicating that nucleotides penetrated into colonic tissue and targeted activated macrophages. In two colitis models, 5 mg/kg intracolonic administered Gal–LMWC–ASO significantly reduced colonic TNF-α mRNA and protein levels. This effect was more prominent compared to naked ISIS 25302. For instance, Gal–LMWC–ASO reduced TNF-α mRNA and protein levels by approximately 60–90% and 50%, respectively, whereas the reduction seen with ISIS 25302 alone was approximately 50% and 10%, respectively. A similar reduction of inflammatory Th1 and Th17 cytokines was observed and this effect was also more prominent for Gal–LMWC–ASO compared to naked ISIS 25302. These results were mirrored by several disease parameters such as mortality, body weight and colonic MPO activity.

Another formulation using ISIS 25302 is GGG-ASO. However, GGG-ASO is a microspheric oral formulation (~650 µm) in which ISIS 25302 is complexed in a glucomannan–gellan gum mixture. Due to this polysaccharide mixture, the formulation has a time-dependent release mechanism targeting the colon. Furthermore, the mannose entities of glucomannan aid in macrophagic phagocytosis of the formulation, which express mannose receptors [115]. In vitro as well as in vivo results indeed showed that the mannose receptor was highly expressed on macrophages, but not colonic epithelial cells and that the formulation therefore was predominantly targeted to colonic macrophages. In colitic mice, oral administration of 50 mg/kg GGG-ASO significantly decreased colonic TNF-α expression by 50%. Significant reductions in other colonic cytokines were observed as well. Additionally, significant effects on mortality, loss of body weight, DAI, colon length, MPO activity, and histological scores were reported.

CAL-ASO is also a formulation using ISIS 25302, which is complexed with lentinan and encapsulated in a chitosan–alginate hydrogel [116]. The complexion with lentinan protects the ASO from degradation while the chitosan–alginate hydrogel yields an oral colon-targeted formulation. In vitro experiments also demonstrated that lentinan increased macrophagic uptake of the formulation and this resulted in reduced TNF-α mRNA and protein expression by 50% and 40%, respectively. Furthermore, in vivo tissue analysis as well as imaging showed that the formulation was targeted to the small intestine and colon. Colonic TNF-α secretion was significantly reduced by 30% and significant effects on loss of body weight, colon length, spleen size, MPO activity, and colonic malondialdehyde (MDA) levels were also observed.

SPG-ASO is an enema containing ASO against TNF-α complexed with the polysaccharide schizophyllan, which is a β-(1–3) glucan [117]. The complexion resulted in a stable ASO formulation that was targeted to cells expressing the Dectin-1 receptor. This pattern recognition receptor is expressed on immune cells and can interact with β-(1–3) glucans to aid in phagocytosis [118]. It was shown that Dectin-1 was significantly upregulated during DSS colitis in mice. Furthermore, SPG-ASO uptake by cells expressing Dectin-1 was significantly increased compared to ASO alone. Rectal administration of 0.2 mg/kg SPG-ASO resulted in significant improvements on body weight, colon length and endoscopic evaluation. Moreover, the expression of TNF-α, IL-1β and IL-6 mRNA was significantly inhibited (~80%) and this effect was the strongest for the SPG-ASO when compared to the rectal administration of the ASO alone.

ASO-miR-301a is an enema containing an ASO against miRNA 301a (miR-301a) [119], which is involved in the pathogenesis of several autoimmune diseases and cancers [120]. Levels of miR-301a were increased in the inflamed mucosa and peripheral blood mononuclear cell of CD and UC patients whereas no increased levels were observed in the unaffected mucosa of these patients. Furthermore, TNF-α expression in CD patients was positively correlated with miR-301a expression in the intestinal mucosa [119,121]. Intracolonic administration of ASO-miR-301a in a TNBS acute colitis model in mice resulted in a significant inhibition of miR-301a expression. This was mirrored by a significant inhibition of TNF-α, IL-17A and RAR-related orphan receptor gamma-t (RORγt) expression—all of which were inhibited by approximately 50%. Additionally, the formulation significantly alleviated colitis symptoms as assessed by DAI, colon length, loss of body weight and histological scores. Phenotype analysis of T cells showed that these anti-inflammatory effects were predominately the result of Th17 cell inhibition. Though the formulation inhibited TNF-α levels locally in the colon, other effects resulting from the interference with different pathways in different tissues cannot be ruled out for miR-301a has different effects in different tissues [120].

### 3.4. microRNA

Gal-LMWC-pre-miR-16 is a formulation containing miR-16 precursor complexed with galactosylated low molecular weight chitosan in view of macrophagic targeting by the MGL [122]. Studies have reported the involvement of miR-16 in IBD and this formulation was therefore investigated [123,124,125]. Intracolonic administration of the formulation corresponding to 5 mg/kg miR-16 targeted TNF-α and IL-12p40 production. The latter is a subunit of the proinflammatory cytokines IL-12 (IL-12p70) and IL-23 and are both involved in IBD [126]. In a TNBS colitis model, the formulation was predominately targeted to colonic macrophages and miR-16 precursor was subsequently metabolized to miR-16. Significant reductions in TNF-α and IL-12p40 mRNA (~50%) as well as protein (~50%) levels were reported. These observations were consistent with the reported significant effects on mortality and disease severity. Comparable anti-inflammatory results in an acute TNBS model have been reported with miR-195, a miRNA also implicated in IBD [127]. However, the latter study did not report a route of administration. Crucially, the authors used a TNBS colitis model for the investigation of UC. To the best of our knowledge, it is uncommon to use this animal model for UC research since the DSS colitis model correlates better with UC [79,80,128].

Of note, the observed anti-inflammatory effects of the investigated miRNA’s may have been partly the result of the miRNA’s interfering with other targets than TNF-α and IL-12p40 expression since both are expressed and regulated in a wide variety of cells [123,125,127,129].

### 3.5. Small Interfering RNA

PACC-siRNA-TACE is an IV formulation consisting of siRNA against TACE complexed in disulfide-linked poly arginine–cysteine complex (PACC) [130]. This biodegradable complex envelops the siRNA, which protects, stabilizes and facilitate targeted cellular uptake. In vitro results showed that PACC significantly increased macrophagic uptake and decreased TACE mRNA levels and TNF-α production compared to siRNA-TACE alone. TNF-α production was inhibited in a dose-dependent manner up to 50%. In an acute colitis mode, IV administration of a dose corresponding to 20 µg siRNA showed that TACE expression was inhibited, resulting in significant reductions of TNF-α (~75%), IL-1β (~75%) and IL-6 (~50%) production. Consistent with these observations were significant reductions in mortality and disease severity as well as effects on the expression of several proteins involved in inflammatory processes. In addition, comparable effects were observed in a chronic colitis model. Taken together, these results show that targeting TACE results in the in vivo inhibition of acute and chronic inflammatory processes. However, this formulation was administered IV and the observed effects may have been partly the result of systemic immune suppression as opposed to localized effects in the colon.

GTC-siRNA is an intracolonic administered formulation consisting of nanoparticles that contain siRNA against TNF-α complexed with galactosylated trimethyl chitosan–cysteine [133]. In macrophages, GTC-siRNA uptake was significantly increased compared to naked siRNA showing that galactosylated trimethyl chitosan–cysteine aids in cellular targeting. Significant in vivo anti-colitic effects on colonic TNF-α mRNA and protein expression, loss of body weight, MPO activity and histology were observed. Noteworthy, experiments on formulation particle size and binding affinity of siRNA for galactosylated trimethyl chitosan–cysteine showed that in vitro macrophagic endocytosis was not dependent on particle size (range 175–450 nm). However, cytoplasmic internalization of siRNA, in vitro epithelial permeability, and in vivo efficacy was dependent on these factors. In general, a formulation with a greater particle size (450 nm) and moderate binding affinity for the siRNA was the most efficacious. Due to the size, these particles were better retained in the colonic lumen whereas the moderate binding affinity assures that the siRNA remains complexed, and therefore, protected from the harsh GI environment while facilitating intracellular release as opposed to a stronger binding affinity. These observations may therefore give guidance in the development of nucleotide formulations intended for the treatment of IBD.

Lipoplex-siRNA-2 is an enema containing liposomal, chemically modified siRNA against TNF-α [135]. Chemically modifying siRNA may increase the silencing capacity, resistance to degradation or both. For instance, a propanediol and double methylation of siRNA at the 3′- and 5′-end, respectively, showed an increased silencing capacity and stability. This siRNA was then formulated to a liposomal enema and administered to colitic mice. Significant reductions in colonic mRNA expression (~40%) as well as mortality, DAI, MPO activity and histological scores were observed. In addition, gene analyses of 25,000 genes showed that 4000 genes were significantly altered after colitis induction. Of these 4000 genes, expression of 60 were significantly altered during Lipoplex-siRNA-2 treatment showing the involvement of TNF-α in a wide range of proinflammatory processes. Comparable effects on TNF-α inhibition and histology has been reported in an earlier study that also investigated an enema containing liposomal siRNA against TNF-α (Lipoplex-siRNA-1) [134].

CycD-siRNA is an enema containing nanoparticles (~240 nm) consisting of siRNA against TNF-α complexed with amphiphilic cyclodextrin [136]. This complexion yields a stable siRNA formulation with good transfection properties [149]. In simulated colonic fluids mimicking fasted and fed state, CycD-siRNA remained stable for up to 24 h. In vitro results showed significant inhibition of TNF-α expression of up to 80%. The effectiveness was investigated in an acute DSS colitis model in mice. These in vivo results showed remarkable improvements in disease severity. Interestingly, TNF-α and IL-6 expression in the proximal colon was significantly reduced whereas IL-6 expression in the distal colon was and TNF-α expression was not significantly inhibited. It has been shown that TNF-α expression in the proximal colon is higher compared to the distal colon in DSS-induced colitis in mice [150]. This may, partly, explain the higher level of gene silencing in the proximal colon, as brought for by the authors [136].

**Figure 3:**
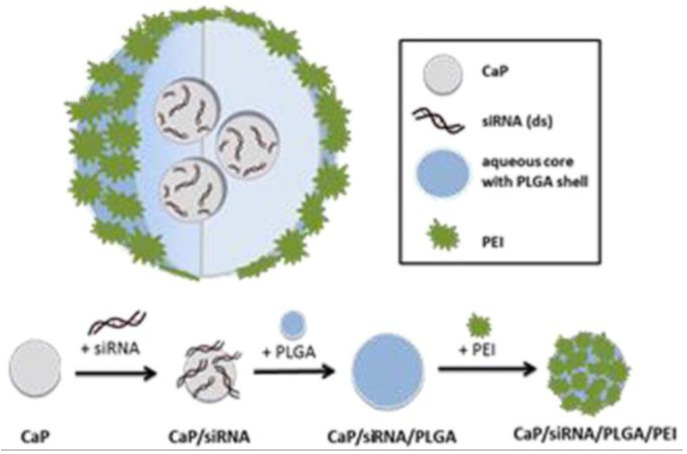
CaP-siRNA is an enema containing nanoparticles (~150 nm) of siRNA against TNF-α loaded on calcium phosphate (CaP), which is then encapsulated in poly(lactic–*co*-glycolic acid) (PLGA) coated with polyethylenimine (PEI). Reprinted from Frede et al. [137] with permission from Elsevier.

CaP-siRNA is an enema containing nanoparticles (~150 nm) of siRNA against TNF-α loaded on calcium phosphate, which is then encapsulated in poly(lactic–*co*-glycolic acid) (PLGA) coated with polyethyleneimine (Figure 3) [137]. Calcium phosphate was used as an siRNA carrier, whereas the PLGA–PEI encapsulation served as a protection mechanism against degradation that targeted and released the siRNA in a controlled manner [151,152,153,154]. The formulation showed a significant reduction in TNF-α expression in vitro. Rectal treatment of colitic mice with 12 µg showed a significant downregulation of colonic TNF-α (~50%), also showing significant effects on loss of body weight, DAI, hematocrit levels and histology scores. Further analyses showed that cellular uptake in the distal colon was the greatest and this uptake was enhanced during colitis, which also shows that the nucleotides could penetrate into colitic tissue [113]. The cells predominantly targeted by CaP-siRNA were colonic dendritic cells, macrophages and T cells, whereas colonic B cells showed minimal uptake.

US-siRNA is an enema containing siRNA against TNF-α which is administered concurrently with rectal 40-kHz ultrasound bursts [138]. Ultrasound can reversibly increase tissue and cellular membrane permeability by a mechanism known as transient cavitation, which facilitates the delivery of oligonucleotides [155,156,157]. Short bursts of 40-kHz ultrasound administered by a rectal probe were safe and well tolerated by colitic mice. Rectal administration of 200 ng of US-siRNA combined with 0.5-s bursts of 40-kHz ultrasound resulted in a significant alleviation of colitis as assessed by fecal and histopathology scores. Proximal as well as distal colonic TNF-α levels were significantly lower (~80%) compared to rectally administered siRNA without ultrasound. Interestingly, ultrasound could also mediate colonic mRNA delivery, which is a bigger macromolecule compared to the mentioned siRNA against TNF-α (950 kDa vs 16 kDa, respectively). Therefore, this method could serve as an approach for the delivery of different nucleotide formulations.

ROS-siRNA is an oral formulation consisting of nanoparticles (~600 nm) containing siRNA against TNF-α encapsulated in a reactive oxygen species (ROS)-sensitive material [139]. The ROS-sensitive encapsulation ensures that the orally administered siRNA is protected against the harsh GI environment, but is released at sites of GI inflammation where ROS concentrations are high [158,159]. For instance, biodistribution analyses showed that colons, but not other tissues of colitic mice had an increased uptake of siRNA compared to healthy mice. Additionally, the tissue-targeting performance of ROS-siRNA was superior compared to a formulation that used a β-glucan encapsulation, which is a suitable method for oral siRNA delivery [160]. Multiple daily doses of ROS-siRNA corresponding to 0.23 mg/kg/day siRNA showed significant improvements in loss of body weight and histology as well as reductions in colonic TNF-α, IL-1, IL-6, IL-12 and INF-γ expression. Together these experiments showed that ROS-siRNA is not only targeted to the colon, but to the inflamed sites of colitic mice. This platform may therefore be used to develop novel therapies targeting the inflamed tissues in IBD.

GalC-siRNA is an oral formulation consisting of nanoparticles (~250 nm) containing siRNA against TNF-α loaded on PLGA after which a galactosylated chitosan layer is added for macrophagic targeting by MGL [140]. In vitro studies showed that the formulation had controlled-release characteristics and was able to protect siRNA against enzymatic degradation in GI homogenates of mice. Moreover, galactosylated chitosan layer indeed increased cellular uptake by macrophages compared to a formulation without galactose modifications. In colitic mice, the formulation significantly reduced TNF-α mRNA (50%) and protein (45%) expression and ameliorated colitis symptoms reflected by the DAI, loss of body weight, colon length, MPO activity and histopathology. Three oral doses ranging from 66–660 µg/kg were administered, but no clear dose–effect relationship was observed.

Nanoparticle-in-microsphere oral delivery system (NiMOS) is also an oral formulation containing nanoparticles (~210 nm) entrapped in microspheres (~3 μm). This system can be used to encapsulate siRNA against TNF-α (NiMOS-siRNA) [141]. After oral administration it remains stable in gastric conditions but releases the content at intestinal pH in the presence of lipases. The effectiveness of this formulation has been investigated in in vivo experiments. These results showed a significant reduction in colonic TNF-α mRNA (60%) and protein (80%) levels in colitic mice treated with 1.2 mg/kg. Expression of several colonic proinflammatory cytokines and chemokines were reduced as well and this resulted in significant clinical effects as assessed by loss of body weight, colon length, MPO activity and histological evaluation. Additionally, the same research group [144] has investigated NiMOS containing a combination of siRNA against cyclin D1 (CyD1) and TNF-α (NiMOS-siRNA-CyD1). CyD1 is a protein involved in cell proliferation [161] and is overexpressed in IBD [162,163]. Similar effects with regards to TNF-α, cytokines, chemokines, histopathology and clinical improvements were observed whether the formulation contained siRNA against only TNF-α or CyD1—or a combination thereof given as a 1.2-mg/kg oral dose. Interestingly enough, the most pronounced effects were seen with the formulation containing only siRNA against CyD1, indicating that CyD1 is involved in key inflammatory processes in IBD.

CA-siRNA is an oral colon-targeted formulation containing nanocomplexes of siRNA against TNF-α encapsulated in chitosan–alginate [142]. This formulation released the nanocomplex in the intestinal environment at a pH 5-6. In an acute inflammation model, mice were first pre-treated orally with 5 mg of the formulation. Subsequently, LPS was administered systemically to induce an acute inflammatory state. Thereafter, TNF-α levels in blood, liver and colon were analyzed. CA-siRNA treatment significantly reduced TNF-α levels in the blood and colon, but not the liver. TNF-α levels in the blood and colon were reduced by 16% and 94%, respectively, demonstrating the effectiveness as well as targeting performance of this formulation.

**Figure 4:**
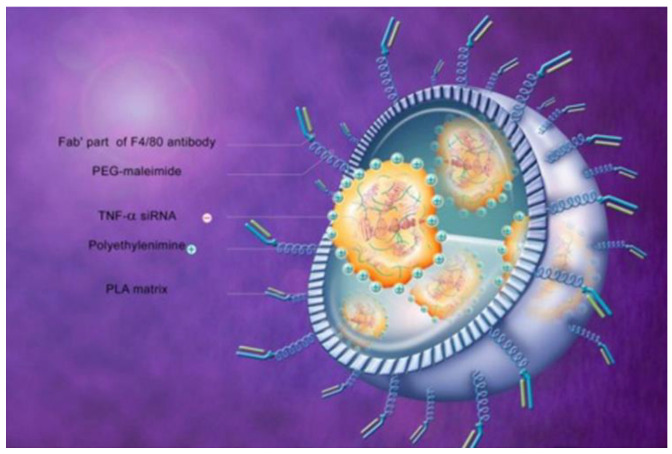
Fab’-siRNA without the chitosan–alginate encapsulation is a nanoparticle of ~375 nm containing siRNA against TNF-α bearing the antigen-binding fragment (Fab’) of the F4/80 antibody, which is specific for murine macrophages. Abbreviations: PLA—polylactic acid; PEG—polyethylene glycol. Reprinted from Laroui et al. [143] with permission from Elsevier.

To increase the targeting performance, the same research group synthesized CA-Fab’-siRNA [143]. This formulation is a nanoparticle containing siRNA against TNF-α enveloped by a surface bearing a covalently bonded antigen-binding fragment (Fab’) of the F4/80 antibody which is further encapsulated in a chitosan–alginate hydrogel for colonic targeting. The F4/80 antibody specifically targets murine macrophages [164]. Figure 4 shows this formulation without the chitosan–alginate hydrogel. This approach was used to target the oral formulation to the colon after which the Fab’ portion specifically interacts with the colonic macrophages, inducing endocytosis and TNF-α mRNA silencing. Several cytotoxicity tests showed that the formulation was biocompatible. Furthermore, in vitro results showed that the formulation preferentially interacted with cells expressing the F4/80 protein and that macrophage endocytosis was increased compared with a formulation without the Fab’ portion. In addition, the formulation significantly reduced the in vitro TNF-α secretion by activated macrophage and the in vivo efficacy in mice was confirmed by substantial reductions in loss of bodyweight, MPO activity and activation of the nuclear factor kappa-light-chain-enhancer of activated B cells (NFκb) pathway as assed by nuclear factor of kappa light polypeptide gene enhancer in B-cells inhibitor-alpha (IκB-α) protein analysis.

Gal-siRNA-IL-22 is an oral formulation as well and consist of nanoparticles containing the cytokine IL-22 and siRNA against TNF-α in galactosylated PLGA, which is further encapsulated in a chitosan–alginate hydrogel [145]. A combination of IL-22 and siRNA against TNF-α was chosen based on data that show that IL-22 possesses mucosal-healing properties [165]. The nanoparticle without the chitosan–alginate encapsulation was ~260 nm and in vitro results showed that macrophagic uptake of the nanoparticle was significantly greater compared to a non-galactosylated formulation. In vitro TNF-α inhibition confirmed these results, showing a significantly increased inhibition of the galactosylated formulation. In vivo biodistribution experiments showed that the formulation was targeted to the colon and that siRNA penetration was the greatest in the mucosa of colitic mice compared to healthy mice, which further shows that nucleotide penetration into colonic tissue is feasible [113,137]. Oral treatment of colitic mice with Gal-siRNA-IL-22 corresponding with 20 μg/kg siRNA and 50 μg/kg IL-22 showed significant improvements in disease severity as assessed by loss of body weight, colon length, spleen weight, histology and MPO activity. Interestingly, colonic TNF-α mRNA expression of colitic mice treated Gal-siRNA-IL-22 did not differ significantly compared to healthy control whereas an increase was seen for mice treated with the same formulation that contained only IL-22 or siRNA against TNF-α. This increased efficacy of the combination therapy was consistent with the other investigated disease parameters, which suggests that a combination of anti- and proinflammatory therapy is superior to either one.

### 3.6. Prokaryotes

Lacto-scFv is the prokaryote *Lactococcus lactis*, subspecies cremoris MG1363, carrying an eukaryotic expression plasmid coding for a single-chain fragment variable (scFv) antibody against TNF-α in view of transforming the epithelial cells of the host [146]. This prokaryote is extensively studied, apathogenic and non-invasive [166]. In an acute colitis model in mice, oral treatment with once daily 2.0–2.5 × 10^9^ colony-forming units (CFU) giving for four days resulted in significant effects on DAI, loss of body weight, colon length, histological scores and CRP. Furthermore, significant downregulation of colonic mRNA expression of TNF-α (~50%) and proinflammatory cytokines was observed. No adverse effects were reported. Taken together, these observations show that Lacto-scFv was able to deliver the eukaryotic plasmid for expression in the host’s cells of the GIT, and thereby, ameliorated colitis in vivo.

In another study investigating the anti-inflammatory effects of engineered *L. lactis*, the exact same strain was modified to secrete bivalent single domain antibody fragments (nanobody) against TNF-α (Lacto-Nanobody) [147]. This nanobody could neutralize sTNF-α as well as tmTNF-α. Oral administration of multiple doses of 2 × 10^9^ CFU resulted in an average of 4 × 10^8^ CFU in the entire colon of colitic mice producing approximately 10 ng nanobody per entire colon. These nanobodies were detected in the mucosa and lamina propria while no nanobodies could be detected in the systemic circulation. Positive effects on histopathologic scores and MPO activity was seen in two chronic colitis models. However, TNF-α, cytokines, inflammatory markers or clinical scores were not analyzed and therefore the effects of this formulation on chronic inflammatory processes is unclear.

### 3.7. Eukaryotes

PRX-106 is an oral formulation containing plant–cell-expressed (BY-2 cell line) recombinant fusion protein of sTNFR2 fused to the Fc part of human immunoglobulin (Ig)G1. The DNA sequence of this protein expressed by the plant cell line is the same as for the commercially available etanercept [148]. In vitro stability studies showed that the protein remained stable during simulated gastric conditions at different pH values. This effect was attributed to the protective effect of the plant cell wall protecting the expressed PRX-106 from the harsh simulated GI environment. In a TNBS acute colitis model, PRX-106 had a significant effect on loss of body weight and improved the histopathology. No colonic levels of TNF-α or cytokines were analyzed and therefore, the local in vivo anti-inflammatory effects of this formulation in this animal model remains unclear. However, this formulation has been investigated in a clinical trial [167] and is discussed in Section 4.

## 4. Clinical Studies on Local TNF-α Inhibition

### 4.1. Considerations

The studies that investigated local TNF-α inhibition in humans were mostly limited and small pilot studies (Table 2). Furthermore, several of these studies were inherently selection biased since patients were included for unconventional therapy due to disease activity that could not be resolved with conventional therapy. In some studies, local therapy was accompanied with surgery, another form of intervention, and/or without a control group. Moreover, the disease type and investigated dose differed as well as the definition of ‘effectiveness’. We therefore chose ‘a favorable clinically relevant response’ as our definition of ‘response’. Due to these limitations, no unambiguous statements about the efficacy regarding local TNF-α inhibition in IBD can be made. However, these data may provide insight into whether local TNF-α inhibition in IBD is feasible, induces a clinically relevant response, and to some extent the mechanism of action of anti-TNF-α therapy in IBD. Of note, research shows an interplay between systemic anti-TNF-α therapy and a favorable response related to the effects on the microbiome [66,67,68,69,168]. Currently it is unclear whether local TNF-α inhibition at the site of inflammation induces these same effects to the same extent. Local TNF-α inhibition generally results in higher drug concentration at the site of inflammation, which is predominantly the gut. This could potentially result in a more prominent effect on the locally present microbes. On the other hand, systemic administration of anti-TNF-α therapy exposes the patient systemically to TNF-α inhibition, and therefore, the overall effect on the microbiome may be more prominent. It would therefore be interesting and valuable to investigate the changes and responses of the microbiome to local TNF-α inhibition compared to systemically administered anti-TNF-α therapies.

### 4.2. Local Injections

In the first study investigating local anti-TNF-α administration in perianal fistulizing CD, a dose of 20 mg IFX divided by several SC injections was administered at the site of inflammation in 9 patients. This study included patients that did not have a sustained response to IV IFX or other systemic drugs. A clinical response was seen in 7 patients of which 4 showed complete healing of the fistulas in 4 weeks. During the 6-month follow-up period no development of ADA was observed [169]. In another study of perianal fistulizing CD investigating 20-mg local IFX injections, 11 patients were included with long-existing luminal disease complicated by perianal disease manifestation. These patients did not respond to conventional treatment such as mesalamine, cortisone, and/or azathioprine. A favorable response was seen in 8/11 patients who received 3–5 injections during an average follow-up of 10.5 months [170]. Similar results with approximately the same doses of local injections at the site of inflammation in perianal fistulizing disease have been reported in two other studies. In these pilot studies [171,172] patients were included who did not respond to or had a contraindication to IV IFX. Furthermore, patient refractory to conventional therapy were included as well. Approximately 60% of the included patient responded to local IFX therapy (range: 2–12 injections). Clinical healing or complete fistula closure was reported in some patients during a follow-up period ranging from 7–43 months. However, one patient was switched to IV IFX after no favorably response was seen during local treatment and developed a delayed hypersensitivity reaction, presumed by the authors as a consequence of local IFX therapy [172]. No major adverse events were reported in the other studies investigating local IFX injections in perianal fistulizing CD [169,170,171].

Local adalimumab injections combined with surgical therapy has also been investigated in perianal fistulizing CD. One study investigating 40-mg injections every 15 days reported complete closure of fistulas in 40% of patients (*n* = 33) after a median number of 9 injections and median follow-up of 11 months. These patients responded without the need for other medical therapy. However, 24% failed to respond to therapy and one adverse event (complication not stated) was reported [174]. Another study reported a response of 100% in 12 patients of which 75% achieved complete cessation of fistula drainage while the remaining patients showed only improvements [175]. The median number of injections for each patient was 7 (range: 4–16) with an average follow-up of 17.5 months. No relapse or adverse events were reported. Similar results with 10-mg local adalimumab injections has been reported elsewhere, showing a response of 100% (*n* = 9) of which resolution of the fistula was observed in 78% of patients after an average therapy period of 23 week [176]. Interestingly, one study investigated the efficacy of local adalimumab injections in perianal fistulizing CD patients that showed no clinical response to local IFX injections. The authors stated that healing of the fistulas was seen in some patients, but did not report definitive success/failure rates nor follow-up periods [173]. A systematic review which included six of the discussed studies [169,170,171,172,174,175] regarding local anti-TNF-α therapy in perianal CD disease reported a partial or complete response rate of 40–100% out of 92 patients included [183].

Endoscopy-guided local IFX injections in CD has been investigated as well. A study with 8 patients investigated the feasibility and effectiveness of IFX injected locally during colonoscopy in localized inflamed regions (<5 cm) [177]. The dose and therapy duration varied, since this depended on the number of inflamed regions as well as the number of colonoscopies. In general, 20–60 mg IFX was administered per colonoscopy, which were several weeks apart (2–4 weeks) with a maximum of three colonoscopies. These patients were concurrently treated with conventional medication and did not relapse (follow-up range: 14–21 months). Although the endoscopy score improved in only 3/8 patients, the number and extent of the inflamed lesions were reduced in 7/8 patients. In another report (*n* = 4) [178] investigating endoscopy-guided IFX injections (20–30 mg) in isolated mucosal lesions, 1 patients showed complete resolution, 2 showed partial improvement and no effect was seen in the last patient. Therapy was variable and ranged from one local injection in total to several injections that were weeks apart. No adverse events were reported in these two studies [177,178].

The effectiveness and feasibility of locally injected IFX in structuring CD has also been reported. One case series (*n* = 3) investigated locally injected IFX (90–120 mg) in colonic strictures [179]. One patient did not respond to chronic IFX infusions, but complete resolution of the stricture was observed after the first local IFX injection. The patient needed a total of two local IFX injections and was symptom free during a follow-up of 5 months. Local IFX injections were effective in the other two patients. However, one patient needed additional manual stricture dilation whereas the other patient needed a total of 5 local injections every 4 months. The patients were followed for 5–8 months and were symptom free during this period. In small bowel strictures of CD, a dose of 40 mg locally injected into the strictures combined with balloon dilation at *t* = 0, *t* = 2 and *t* = 4 weeks showed a response of 100% in 6 patients. No adverse events or relapses were reported during a follow-up period of 6 months [181]. In addition, balloon dilation combined with locally injected IFX (25 mg) showed clinical improvements in anal stenoses of CD (*n* = 2) [180]. However, one patient needed a total 16 injections in a period of approximately 6 years.

### 4.3. Topical Treatment

In our search we found that topical anti-TNF-α therapy has only been investigated in one case report regarding a UC patient [182]. The patient suffered from refractory pancolitis not responding to conventional therapy. Therefore, subtotal colectomy and ileorectal anastomosis took place. Despite conventional therapy including IV IFX, severe symptoms remained. Symptoms improved after instillation of daily 100-mg IFX enemas for 6 days. Clinical and histological scores improved without any reported adverse events.

### 4.4. Oral Therapy

PRX-106 is an oral formulation containing plant–cell-expressed (BY-2 cell line) recombinant fusion protein of soluble TNF receptor 2 (sTNFR2) fused to the fragment crystallizable region (Fc) part of human IgG1. This formulation is also discussed in Section 3. [148]. A phase I clinical study [167] in 14 healthy volunteers investigated daily oral doses corresponding with 2 mg, 8 mg and 16 mg of active compound for 5 days. Analyses of different T cell subtypes showed minimal inhibitory or stimulatory effects on the differentiation of these cell types. In general, no remarkable effects on TNF-α, IFN-γ IL-4, IL-10 and IL-12 were observed. Additionally, no clear dose–response effect was reported. The treatment-related adverse events were mild. To date, it is unclear whether this specific formulation has an anti-inflammatory effect or is efficacious in the treatment of IBD patients. However, IBD treatment with SC etanercept is not efficacious in active CD [184].

AVX-470 is an orally administered antibody against TNF-α and is also discussed in Section 3. [105]. The efficacy and safety in active UC patients has been investigated in a double-blind, placebo-controlled dose-finding study [107,108]. Patients (*n* = 37) with an established diagnosis of UC with colonic involvement were enrolled of which approximately 50% had a history of pancolitis. Treatment consisted of 0.1 g, 0.78 g or 1.17 g of oral AVX-470 twice daily for 28 days. Therapy was well tolerated in all three treatment arms with no serious drug-related incidents. Pharmacokinetic analysis showed no substantial systemic AVX-470 absorption or the development of antibodies against bovine Ig, which was a surrogate marker for the immunogenicity of AVX-470. However, stool bovine Ig increased with increasing dose suggesting that AVX-470 transited through the entire GIT, which is consistent with the observed GI stability of orally administered Ig [77,78]. Pharmacodynamic analyses were considered exploratory due to the relatively small study population and short duration of therapy (4 weeks). Nonetheless, a trend towards a dose-dependent relationship was seen for clinical remission, endoscopic response and endoscopic remission. In the 3.5-g/day treatment arm, 14% of patients reached clinical remission as well as endoscopic remission compared to 0% in the placebo group (not powered for statistical significance). Furthermore, favorable responses in Mayo scores and serum CRP and IL-6 were observed. Interestingly, the same research group analyzed colonic biopsies of the included patients in a separate study [107] and found that clinical response was correlated with a reduction in tissue TNF-α levels and other inflammatory markers. Moreover, it was observed that bovine Ig penetrated the submucosal tissue of UC patients whether there was visible inflammation or not. However, bovine Ig did not penetrate into the tissue of healthy volunteers. Of note, bovine Ig was a surrogate marker for AVX-470, but these results could have been confounded by the consumption of dairy products or beef. Nonetheless, it shows that an antibody can penetrate into the inflamed mucosa and submucosa in UC patients. This has also been reported in several colitis animal studies [92,105,109], which is consistent with this observation.

V565 is a 115 amino acid 12.6 kDa single domain antibody and is also discussed in Section 3. [92,94]. Results from a clinical trial [104] showed that active V565 could be recovered in the ileal fluids of patients with a terminal ileostomy after oral administration of enteric-coated tablets with a pH threshold ≥ 6. In CD patients, high active V565 concentrations could be recovered in fecal samples. However, serum levels could not be detected. In UC patients, V565 penetrated the lamina propria and inhibited the phosphorylation of several proteins involved in inflammatory responses. No serious adverse events were reported. Though no formal efficacy study was performed, these results show that V565 is stable during GI transit, can penetrate into the lamina propria of UC patients and exert a pharmacological effect. This result, together with observations of AVX-470 [107,108] and animal studies [92,105,109], corroborate the theory that orally administered antibodies penetrate into the inflamed tissues in IBD and exert a pharmacological effect.

## 5. Conclusions

Local TNF-α inhibition in IBD is feasible. Animal studies showed that topical antibody or nucleotide therapy could penetrate into the inflamed sites of the GIT and exert a pharmacological effect. This observation was consistent for antibodies that were investigated in clinical studies.

Experimental animal studies showed the feasibility and efficacy of local TNF-α inhibition in IBD models. Nonetheless, several challenges remain for these experimental therapies due to the relatively unconventional mechanism of action. For instance, appropriate dose regimens, dose escalation algorithms, and mechanisms to terminate oligonucleotide as well as prokaryotic therapy should be available before these therapies reach the clinical settings. Furthermore, methods to evaluate the site-specific targeting performance of oligonucleotide formulation are of great value since mucosal concentrations may not always correlate with the intracellular drug concentrations of the targeted cells.

The clinical studies showed the effectiveness of local TNF-α inhibition realized by local injections, topical treatment or oral therapy. For some patients not responding to conventional therapy, which included systemic anti-TNF-α therapy, local TNF-α inhibition was effective. However, most clinical studies were inherently limited, biased, and used a patient unfriendly route of administration. A prerequisite for adequate therapy and patient compliance is a patient friendly formulation with the objective to inhibit TNF-α locally. An effective oral formulation targeted to the site of inflammation meets these requirements. Several formulations discussed in this review aim to achieve this treatment approach.

The different approaches discussed in this review aimed to target the anti-inflammatory therapy to the site of inflammation in view of maximizing the local efficacy while minimizing the systemic effects. Appropriately designed clinical trials are necessary to investigate the efficacy of this approach in IBD. Additionally, it would be interesting to compare local TNF-α inhibition to gold standard anti-TNF-α therapies in these trials. These data would give insight into the efficacy and feasibility of local TNF-α inhibition as well as the mechanism of action of these compounds and TNF-α in IBD.

## Figures and Tables

**Table 1:** Summary of the animal studies investigating the local effects of anti-TNF-α therapy. Abbreviations: AAT—alpha 1-antitrypsin; ASO—antisense oligeonucleotide; DAI—disease activity index; DSS—dextran sodium sulfate; Fab’—antigen-binding fragment; Fc—fragment crystallizable region; GM-CSF—granulocyte-macrophage colony-stimulating factor; H&E—hematoxylin and eosin; IC—intracolonic administration; ICH—immunohistochemistry; IFN-γ—interferon-gamma; IFX—infliximab; Ig—immunoglobulin; IκB-α—nuclear factor of Kappa light polypeptide gene enhancer in b-cells inhibitor-alpha; IP—intraperitoneal injection; IV—intravenously administered; Ly6 g—lymphocyte antigen 6 complex; MCP-1—monocyte chemoattractant protein 1; MIP-1α—macrophage inflammatory protein 1-alpha; MDA—colonic malondialdehyde content; miR—microRNA; MPO—myeloperoxidase activity assay; NADPH—nicotinamide adenine dinucleotide phosphate oxidase activity; NS—not stated; PLGA—poly(lactic–*co*-glycolic acid); PO—orally administered, per os; pSer32/Ser36—phosphorylated serine-32/serine-36; Rec.—rectally administered; ROA—route of administration; ROS—reactive oxygen species; SC—subcutaneous injection; siRNA—small interfering RNA; TACE—tumor necrosis factor-α-converting enzyme; TNBS—trinitrobenzenesulfonic acid; TGF-β—transforming growth factor-beta; TNF-α—tumor necrosis factor-α; scFv—single-chain variable fragment; sTNFR2—soluble TNF receptor 2.

Treatment	Formulation	ROA	Animal Model	TNF-α ^a^	Cytokines ^a^	Measured Effects	Histology	Reference
**Antibodies**								
IFX-Enema	Enema containing an IFX solution	Rec.	Mice, DSS acute colitis	–	–	Body weight, colon length, DAI, Rachmilewitz score	H&E staining	[90]
V565	Anti-TNF-α single domain antibody	PO	Mice, DSS acute colitis	–	–	–	–	[92]
V565 tablet	Anti-TNF-α single domain antibody coated with pH sensitive polymer (pH threshold ≥6)	PO	Cynomolgus monkeys, healthy	–	–	–	–	[94]
Avian-anti-TNF-α	Avian antibody against TNF-α	PO	Rats, TNBS acute colitis	–	–	Colon morphology, colon weight, MPO	H&E staining, histopathology score, IgY staining	[109]
			Rats, TNBS chronic colitis	–	–	Colon morphology, colon weight, MPO	Histopathology score, IgY staining	
AVX-470	Bovine colostral antibody against TNF-α	PO	Mice, DSS acute colitis	–	–	Endoscopy score	–	[105]
			Mice, DSS chronic colitis	mRNA	IL-1β, IL-6, IL-12p40	Endoscopy score, colon length, colon weight, IHC score	Histopathology score, IHC staining	
			Mice, TNBS acute colitis	–	–	Endoscopy score	–	
**Antisense Oligonucleotides**								
ISIS 25302	ASO against TNF-α	SC	Mice, DSS chronic colitis	Protein, mRNA	–	DAI	Histopathology score	[110]
ISIS 25302	ASO against TNF-α	IP	Mice, *db/db*	mRNA	–	-	–	[112]
		IV	Mice, DSS acute colitis	–	–	Colon length, DAI	–	
		SC	Mice, DSS chronic colitis	Northern blot	–	DAI	Histopathology score	
			Mice, IL-10^−/−^ colitis prophylaxis	Colon organ culture, basal and LPS stimulated	–	–	Histopathology score	
			Mice, IL-10^−/−^ colitis therapy	Colon organ culture, basal and LPS stimulated	IFN-γ	–	Histopathology score	
Gal–LMWC–ASO	Nanocomplex of ASO against TNF-α (ISIS 25302) associated with galactosylated low molecular weight chitosan	IC	Mice, TNBS acute colitis	Protein, mRNA	IFN-γ, IL-1β, IL-6, IL-12, IL-17, IL-23,	AAT, body weight, DAI, mortality, MPO	H&E staining, histopathology score, TNF-α staining	[113]
			Mice, CD4^+^ CD45RB^hi^ chronic colitis	Protein, mRNA	IFN-γ, IL-1β, IL-6, IL-12, IL-17, IL-23	AAT, body weight, DAI, mortality, MPO	H&E staining, histopathology score. TNF-α staining	
GGG-ASO	Colon-targeted microspheres containing ASO (ISIS 25302) against TNF-α complexed with a mixture of glucomannan-gellan gum	PO	Mice, DSS acute colitis	Protein	IL-1β, IL-6, IL-12p70, IL-23	Body weight, colon length, DAI, mortality, MPO	H&E staining, histopathology score	[131]
CAL-ASO	ASO against TNF-α (ISIS 25302) complexed with lentinan encapsulated in chitosan–alginate	PO	Mice, DSS acute colitis	Protein	–	Body weight, colon length, MDA, MPO, spleen size	–	[116]
SPG-ASO	Enema containing schizophyllan–ASO complex against TNF-α	Rec.	Mice, DSS acute colitis	mRNA	IL-1β, IL-6	Body weight, colon length, endoscopy	H&E staining, histopathology score	[117]
ASO-miR-301a	Enema containing ASO against miR-301a	IC	Mice, TNBS acute colitis	mRNA	IFN-γ, IL-4, IL-10, IL17A	Body weight, colon length, DAI	H&E staining, histopathology score	[119]
**MicroRNA**								
Gal-LMWC-pre-miR-16	Precursor of miR-16 complexed with galactosylated low molecular weight chitosan	IC	Mice, TNBS acute colitis	Protein, mRNA	IFN-γ, IL-1β, IL-6, IL-12p40, IL-17A, IL-23	Body weight, DAI, mortality, MPO	H&E staining, histopathology score, IL-12p40 staining, TNF-α staining	[122]
miR-195	Agomir of miR-195	NS	Rats, TNBS acute colitis	Protein, mRNA	IL-1β, IL-6	DAI	H&E staining	[132]
**Small interfering RNA**								
PACC-siRNA-TACE	Poly arginine–cysteine complex containing siRNA against TACE	IV	Mice, DSS acute colitis	Protein	IL-1β, IL-6	Body weight, colitis score, colon length, mortality, MPO, NADPH	H&E staining, histopathology score	[130]
			Mice, DSS chronic colitis	–	–	Body weight, colitis score, mortality	H&E staining, histopathology score	
GTC-siRNA	Nanoparticle containing siRNA against TNF-α complex with galactosylated tri-methyl-chitosan–cysteine	IC	Mice, DSS acute colitis	Protein, mRNA	–	Body weight, MPO	H&E staining	[133]
Lipoplex-siRNA-1	Enema containing liposomal siRNA against TNF-α	Rec.	Mice, DSS acute colitis	mRNA	–	–	H&E staining	[134]
Lipoplex-siRNA-2	Enema containing liposomal siRNA against TNF-α	Rec.	Mice, DSS acute colitis	mRNA	Gene analysis of 25,000 genes	DAI, mortality, MPO, weight-over-length ratio colon	H&E staining, histopathology score	[135]
CycD-siRNA	Enema containing nanocomplex of cationic cyclodextrin complexed with siRNA against TNF-α	Rec.	Mice, DSS acute colitis	mRNA	IL-6	Body weight, colon length, colon weight	–	[136]
CaP-siRNA	Enema containing nanoparticles of siRNA loaded on calcium phosphate and encapsulated in PLGA	Rec.	Mice, DSS acute colitis	Protein, mRNA	–	Body weight, colon length, DAI, hematocrit	H&E staining, histopathology score	[137]
US-siRNA	Enema containing siRNA against TNF-α delivered by ultrasound	Rec.	Mice, DSS acute colitis	Protein	–	Fecal score	Histopathology score	[138]
ROS-siRNA	Nanoparticle containing siRNA against TNF-α encapsulated in a ROS-sensitive polymer	PO	Mice, DSS acute colitis	Protein, mRNA	IFN-γ, IL-1, IL-6, IL-12	Body weight, MPO	H&E staining	[139]
GalC-siRNA	Galactosylated chitosan-coated nanoparticle containing siRNA against TNF-α loaded on PLGA	PO	Mice, DSS acute colitis	Protein, mRNA	IFN-γ, IL-6	Body weight, colon length, DAI, MPO	H&E staining	[140]
NiMOS-siRNA	Nanoparticle in microsphere containing siRNA against TNF-α	PO	Mice, DSS acute colitis	Protein, mRNA	GM-CSF, IFN-γ, IL-1β, IL-2, IL-5, IL-6, IL-12p70, MCP-1, MIP-1α	Body weight, colon length, MPO	H&E staining	[141]
CA-siRNA	Colon-targeted nanoparticle containing siRNA against TNF-α encapsulated in chitosan–alginate	PO	Mice, LPS-induced acute inflammation	Protein	–	–	–	[142]
CA-Fab’-siRNA	Colon-targeted nanoparticle containing siRNA against TNF-α bearing Fab’ of F4/80 antibody encapsulated in chitosan–alginate	PO	Mice, DSS acute colitis	–	–	Body weight, IκB-α, MPO	Ly6 g staining	[143]
NiMOS-siRNA-CyD1	Nanoparticle in microsphere containing siRNA against TNF-α and CyD1	PO	Mice, DSS acute colitis	Protein, mRNA	CyD1, GM-CSF, IFN-γ, IL-1α, IL-1β, IL-2, IL-5, IL-6, IL-17, MCP-1, MIP-1α	Body weight, colon length, MPO	H&E staining	[144]
Gal-siRNA-IL-22	Nanoparticle containing IL-22 and siRNA against TNF-α in galactosylated PLGA encapsulated chitosan–alginate hydrogel	PO	Mice, DSS acute colitis	mRNA	–-	Body weight, colon length, MPO, spleen weight	H&E staining, histopathology score	[145]
**Prokaryotes**								
Lacto-scFv	*Lactococcus lactis* carrying eukaryotic vector coding for a scFv anti-TNF-α	PO	Mice, DSS acute colitis	mRNA	IL-1β, IL-6, IL-10, IL-17A, TGF-β	Body weight, colon length, CRP, DAI	H&E staining, histopathology score	[146]
Lacto-Nanobody	*Lactococcus lactis* secreting bivalent nanobodies against TNF-α	PO	Mice, DSS chronic colitis	–	–	-	H&E staining, histopathology score	[147]
		PO	Mice, IL-10^−/−^, chronic colitis	–	–	MPO	H&E staining, histopathology score	
**Eukaryotes**								
PRX-106	Plant-cell expressed anti-TNF-α fusion protein consisting of sTNFR2 fused to human Fc of human IgG1	PO	Mice, TNBS acute colitis	–	–	Body weight	H&E staining, histopathology score, IκB-α pSer32/Ser36 staining	[148]

^a^: specifically designates (protein, mRNA or both) measured in the gut from in vivo experiments unless otherwise stated.

**Table 2:** Summary of the clinical studies investigating local TNF-α inhibition. Abbreviations: ADA—anti-drug antibodies; IFX—infliximab; NA—not applicable; NS—not stated; PK—pharmacokinetic; PO—orally administered, per os.

Disease	Drug	ROA	Dosage	Therapy	Follow-up	Response ^a^	Remarks	Reference
CD, perianal fistulas (n = 9)	IFX	Local injection	20 mg	0, 1, 3 weeks	6 months	78%	ADA did not develop during follow-up	[169]
CD, perianal fistulas (n = 15)	IFX	Local injection	15–21 mg	0, 4, 8, 12, 16, 20 weeks	18.2 (3–30) months ^b^	67%	Combined with surgical treatment; included nonresponders to IV IFX and patients with contraindication for IFX	[171]
CD, perianal fistulas (n = 11)	IFX	Local injection	20 mg	Every 4–16 weeks	10.5 (7–18) months ^b^	73%	Including patients not responding to conventional systemic therapy	[170]
CD, perianal fistulas (n = 12)	IFX	Local injection	20–25 mg	Every 4–6 weeks	35 (19–43) months ^c^	88%	Combined with surgical treatment; included nonresponders to IV IFX	[172]
CD, perianal fistulas (n = 16)	adalimumab	Local injection	40 mg	Every 15 days	NS	NS, but response was observed	Combined with surgical treatment and including patient who did not respond to local IFX therapy	[173]
CD, perianal fistulas (n = 33)	adalimumab	Local injection	40 mg	Every 15 days	11 (7–14) months ^c^	40%	Combined with surgical treatment	[174]
CD, perianal fistulas (n = 12)	adalimumab	Local injection	20 mg	Every 2 weeks	17.5 (5–30) months ^b^	100%	Including surgical therapy	[175]
CD, perianal fistulas (n = 9)	adalimumab	Local injection	10 mg	Every 2 weeks	NS	100%	Investigated different T cell phenotypes in peripheral blood and fistulas	[176]
CD, postoperative localized recurrent (n = 8)	IFX	Local injection	8–60 mg	variable	20 (14–21) months ^c^	38%	Endoscopy-guided injections into localized regions of <5 cm	[177]
CD, isolated symptomatic regions (n = 4)	IFX	Local injection	20–30 mg	variable	NS	75%	Endoscopy-guided injections into local regions	[178]
CD, colonic strictures (n = 3)	IFX	Local injection	90–120 mg	variable	5–8 months	100%	Manual dilation in 1 patient	[179]
CD, rectal stenosis (n = 2)	IFX	Local injection	25 mg	variable	NS	100%	Combined with balloon dilation	[180]
CD, small bowel strictures (n = 6)	IFX	Local injection	40 mg	0, 2, 6 weeks	6 months	100%	Combined with balloon dilation	[181]
UC, refractory proctitis (n = 1)	IFX	Enema	100 mg	6 days	NS	100%	Patient with subtotal colectomy and ileorectal anastomosis	[182]
Healthy volunteers (n = 14)	PRX-106	PO	2–16 mg	5 days	10 days	NA	PRX-106 was not systemically absorbed and no clear in vivo effects were seen	[167]
UC, colonic involvement (n = 37)	AVX-470, capsule	PO	0.2–3.5 g	4 weeks	7 weeks	14% ^d^	Colonic biopsies were analyzed in a separate study [107]	[108]
CD (n = 6)	V565, enteric coated tablet	PO	555–1665 mg	Single dose	No follow-up	NA	PK study	[104]
UC (n = 5)			1110 mg	7 days			Tissue penetration study	
Non–CD Ileostomy (n = 4)			1665 mg	Single dose			Ileal fluid recovery study	

^a^—specifies ‘a favorable clinically relevant response’; ^b^—mean (range); ^c^—median (range); ^d^—as assessed by clinical remission, endoscopic response and endoscopic remission vs. 0% for the placebo group.
